# Why Do You Make Us Feel Good? Correlates and Interpersonal Consequences of Affective Presence in Speed-dating

**DOI:** 10.1002/per.1944

**Published:** 2013-11-21

**Authors:** RAUL BERRIOS, PETER TOTTERDELL, KAREN NIVEN

**Affiliations:** 1 University of SheffieldSheffield, UK; 2 University of ManchesterManchester, UK

**Keywords:** affect, romantic interaction, individual differences, relationships, attraction

## Abstract

Recent research indicates that people consistently make others feel a certain way (e.g. happy or stressed). This individual difference has been termed affective presence, but little is known about its correlates or consequences. The present study investigated the following: (i) whether affective presence influences others' romantic interest in a person and (ii) what types of people have positive and negative affective presence. Forty volunteers took part in a speed-dating event, during which they dated six or seven opposite-sex partners. A Social Relations Model analysis confirmed that individuals prompted consistent positive emotional reactions in others. Participants were more likely to want to see dates with greater positive affective presence again in the future, and positive affective presence explained the effects of perceived responsiveness on romantic interest. Associations between positive affective presence and trait predictors, including emotion regulation, emotional expressiveness, attachment style, agreeableness and extraversion, were also observed. The findings indicate that what emotionally distinguishes one individual from another lies in part in the emotional consequences of their behaviours on others. © 2013 The Authors. European Journal of Personality published by John Wiley & Sons Ltd on behalf of European Association of Personality Psychology.

## Introduction

Describing someone's personality using terms that pertain to the emotions that the person typically expresses is commonplace. For example, we might refer to someone we know as a happy, relaxed or miserable person. But do we consistently elicit particular emotions in other people? Personality psychologists increasingly believe that ‘personality (how people are) is inseparably interwoven with social relationships (who people are with)’ (Back, Baumert, *et al*., 2011, p. 90), and a recent study suggested that a meaningful amount of variation in how people feel can be attributed to their interaction partner (Eisenkraft & Elfenbein, 2010). The idea that people have a stable *affective presence* when interacting with different people suggests that the emotional consequences of our behaviour on others contribute to make us unique, providing new research avenues to study how personality impacts relationships. In the present study, we investigated whether affective presence influences other people's interest towards a person in a romantic context (a speed-dating event). We also explored personality correlates of affective presence to provide insight into characteristics that predict the emotions that people elicit in others.

### Affective presence

Affective states, such as emotions and moods, are typically conceptualized as short-term states that fluctuate over time (Frijda, 1993). However, results from *in-situ* diary studies that have sampled these fluctuations suggest that there is a trait component to affect (e.g. being generally cheerful or miserable), such that at least some of the variance in people's day-to-day affect is explained by their personality (e.g. Fisher, 2000). To date, most of the evidence on intra-individual stability in affect has focused on the consistency of individuals' own affective states, with evidence suggesting that the affect people experience tends to be relatively stable over time (Watson & Walker, 1996). However, in recent years, there has been increasing attention paid to the social aspects of emotion, and the idea that people can elicit emotional reactions in others (Hareli & Rafaeli, 2008; Hatfield, Cacioppo, & Rapson, 1994; Parkinson, 1996). From the standpoint of individual differences in affect, this raises the question of whether there may be stable individual differences in the emotions that people elicit in others, referred to herein as *affective presence* (Eisenkraft & Elfenbein, 2010), which are independent of people's own experienced emotions. The idea that people might leave a consistent emotional footprint on the various others they interact with derives from several theoretical perspectives, including informational models of emotion (Forgas, 1994; Schwarz & Clore, 1983; Van Kleef, 2009), and theories that emotions are reciprocal and possess communicational influence between people (Keltner & Haidt, 1999; Niedenthal & Brauer, 2012). The idea also shares similarities with Buss's (1987) notion of *evocation*, according to which others' behaviour can be consistently elicited by some individuals.

Despite clear theoretical grounding, only one empirical study to date has provided evidence of affective presence. The study, by Eisenkraft and Elfenbein (2010), involved Master of Business Administration students who were organized into small groups of four to five people. At the end of a month in which every group worked together on a project and socialized regularly, participants evaluated how much they felt eight affective states during their interactions with each of the rest of the members of their work group. Eisenkraft and Elfenbein's findings showed that over and above trait affect and any emotion contagion effects, some people consistently elicited the same positive or negative emotions in others. Affective presence explained 23% of the variance in other people's negative feelings and 10% of the variance in other people's positive feelings (Eisenkraft & Elfenbein, 2010).

Eisenkraft and Elfenbein's (2010) study served to establish the construct of affective presence and has opened up some interesting research questions that could contribute towards recent efforts to integrate personality and social relationships into a unified framework (Back *et al*., 2011; Graber, Laurenceau, & Carver, 2011; Reis, Capobianco, & Tsai, 2002; Zayas, Shoda, & Ayduk, 2002). One question of interest regards the interpersonal consequences of affective presence particularly whether affective presence can facilitate or inhibit the development of new relationships, for example, in romantic contexts. Another question concerns which types of personality characteristics might explain whether a person has a positive or negative affective presence.

### The present study

The aims of the present study were threefold. Our first aim was to investigate whether affective presence influences romantic interest during a speed-dating event. Many theoretical accounts of emotion assert that a primary function of emotion is to regulate interpersonal distance (e.g. Parkinson, 1996). As such, a likely key function of affective presence will be its involvement in the initiation and development of relationships. Emotional expression is thought to boost attraction (Kashdan, Volkmann, Breen, & Han, 2007; Tracy & Beall, 2011), and the pleasure experienced during an interpersonal encounter influences judgments about the desire for future interactions with the other person (Sunnafrank, 1986). Yet, we know little about whether affective presence influences romantic interest in new potential relationship partners.

According to the affect-as-information theory (Schwarz & Clore, 1983), people draw on the way they feel as a source of information about the value of the person with whom they are interacting. Ajzen (1974) further suggested that the affective value of the information provided by strangers predicts the levels of attraction felt by the people who meet them. Likewise, it is expected that interacting with a person who has a positive affective presence would elicit pleasant feelings leading to romantic interest, which will be expressed in the form of an intention to initiate a potential relationship, whereas a negative affective presence would produce the opposite effect. Some support for this proposition is provided by Eisenkraft and Elfenbein's (2010) original study, in which they reported a relationship between affective presence and centrality in the social networks of their participants, indicating that those with more positive affective presence were more popular. However, network centrality provides only an indirect measure of attraction because a person might be friends with someone for a range of reasons other than liking. Consequently, we tested whether affective presence predicts romantic interest during dyadic interactions.

We further explored whether affective presence mediates the effects of a common predictor of interpersonal attraction, which is perceived responsiveness. Perceived responsiveness refers to individuals' perceptions that a relationship partner has attended to and reacted supportively to them (Reis, Clark & Holmes, 2004) and has been consistently related to liking (Davis & Perkowitz, 1979; Miller & Berg, 1984; Reis, Maniaci, Caprariello, Eastwick, & Finkel, 2011). Classical models, such as the affect-reinforcement model of attraction (Clore & Byrne, 1974), have suggested that affect is a mediator in the attraction process. Similarly, we further suggest that the personality characteristic of consistently eliciting positive affect in others may be prompted by the effect of responsiveness. Responsiveness provides the communicational features necessary to activate the partner's personal repertoire to make others feel good. Actually, Davis and Perkowitz (1979) argue that communication of affect is a key function of responsiveness that increases attraction. Consequently, responsiveness heightens perception of this personal repertoire deployment, which ultimately yields generalized affect in others and influences romantic interest. This assertion is in line with recent evidence, which indicates that mate choices can be influenced by personality characteristics (Back, Penke, Schmukle, Sachse, Borkenau & Asendorpf, 2011). As a consequence, we hypothesized that:

*H1:* Affective presence will predict romantic interest during speed-dating, such that positive affective presence will be positively related to romantic interest and negative affective presence negatively related to romantic interest.

*H2:* Affective presence will mediate the association between perceived responsiveness and romantic interest.

Our second aim was to provide insight into the types of personality characteristics associated with positive and negative affective presence, by exploring a range of emotional skills and dispositions in relation to affective presence. In their original study, Eisenkraft and Elfenbein (2010) investigated links between the Big-5 personality traits and affective presence. Here, we extend this by exploring additional variables that we think are likely to be correlates of affective presence, in particular those that pertain to people's communication of emotions and the way that they approach interactions. We venture that affective presence is about the emotions that individuals elicit in others so it is likely to depend on an individual's ability to control and communicate emotions, and it is measured by the reaction of others so it is also likely to depend on how an individual manages interpersonal interactions.

**Figure 1:**
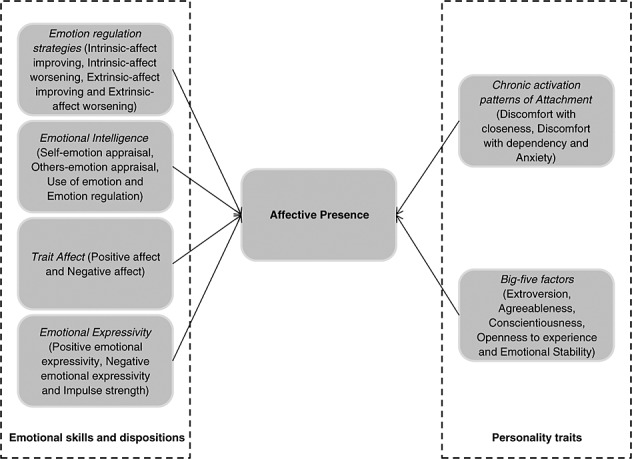
Hypothesized individual difference predictors of affective presence.

Among the correlates of affective presence, we included variables relating to individual differences in how people typically feel (i.e. trait affect) and express (i.e. expressivity) emotions, their emotion abilities (i.e. emotional intelligence), their use of strategies to manage emotions (i.e. emotion regulation) and their emotional distance and anxiety when forming social relationships (i.e. adult attachment style). This set of potential correlates not only allows us to detect which types of people are most likely to have positive and negative affective presence, but also enables insight into whether affective presence arises more from relational, other-oriented characteristics and abilities (in which case, it should be associated more strongly with expressivity, other-oriented emotion abilities and regulatory strategies and attachment style) or from personal, self-related characteristics and abilities (in which case, it should be associated more strongly with trait affect and self-oriented emotion abilities and regulatory strategies). Our selection of correlates of affective presence as other-oriented and self-related draws upon models which also characterize the self either as representations of relational scripts or how the self is in relation with others (e.g. relational-schemas. Baldwin, 1992) or as a collection of knowledge and experiences that help us to organize and anticipate events *via* self-inference (e.g. Markus, 1977). The full set of individual difference variables that we tested is shown in Figure 1.

**Figure 2:**
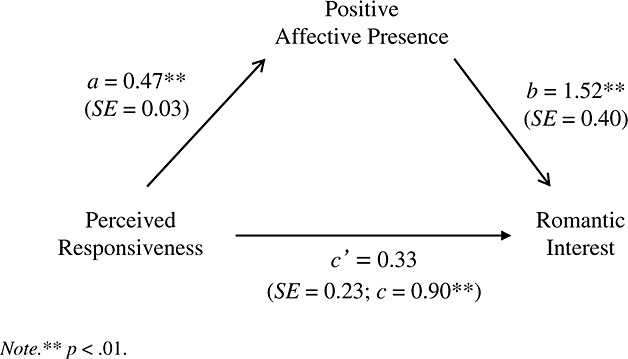
Mediation analysis of the relationship between perceived responsiveness and romantic interest (measured as the desire to see the person again) as mediated by positive affective presence.

Our third aim was to evaluate the robustness of the affective presence phenomenon by using a methodological design that overcomes some of the limitations of Eisenkraft and Elfenbein's (2010) study, and applying this phenomenon to a different social context. We expected that evidence for the existence of affective presence would be replicated by measuring for it immediately after brief romantic interactions (*dates*).

We addressed our three aims using a heterosexual speed-dating event, whereby participants had a series of brief one-on-one dates with other attendees (fellow participants) in search of a potential romantic partner. This method, which has been used by other researchers (Finkel & Eastwick, 2008), provides several advantages in examining affective presence. Specifically, its use of discrete interactions allows the collection of concurrent measures of affective reactions while individuals interact with others, and its sequential dyadic nature helps to reduce the potential influence of confounding variables that can arise from observing individuals within a group. In addition, there is evidence to support the ecological validity of the speed-dating technique to assess interpersonal variables (Asendorpf, Penke, & Back, 2011; Finkel & Eastwick, 2008; Finkel, Eastwick, & Matthews, 2007), like romantic interest.

## Method

### Participants

Forty students (20 women and 20 men, *M_age_* = 25.31 years; *SD* = 3.20 years) participated in the study in exchange for a small discount on a drink purchase and the opportunity to win vouchers worth £35 (approximately €41). Participants were recruited from a convenience sample and were allocated to the study on a first-come, first-served basis. The majority of the participants were native English speakers (62%) and postgraduate students (61%). No participant dropped out of the study but a few had sporadic missing data in the before-event questionnaire responses; so, our sample size varies across analyses for correlates of affective presence.

### Procedure

After informed consent was obtained, participants received a set of online questionnaires a week prior to the speed-dating event. Once completed, participants were assigned to one of three different groups quasi-randomly, so that two groups had 14 participants and one group had 12 participants, with an equal number of men and women in each. The speed-dating was organized on the premises of the University's bar to convey a comfortable space suitable for the romantic tone of the event. Participants were instructed to not drink alcohol for at least 5 hours before the event. On arrival, participants were given identification numbers to allow their dates to identify them on reports during the event. In each group, men were seated in one row and women in the opposite row separated by a table. Each date took 4 minutes and a signal to start and finish each date was sounded by research assistants in each group, who immediately after each date gave participants the questionnaire containing the affective presence measure, the perceived responsiveness scale and the romantic interest question.

Thus, individuals had between six and seven dates during the event, depending on whether they belonged to a table with 12 or 14 participants. This produced a total of 134 dates and 268 observations in total. All participants' personal details were saved in a separate file to assure confidentiality, and participants were fully debriefed about the purpose of this research following the speed-dating event.

### Measures

Data were collected in three stages: in the days before the speed-dating event, after each date during the event and at the end of the event.

#### Stage 1: Pre-event questionnaire

In the pre-event questionnaire, we measured a range of individual differences as potential correlates of affective presence.

##### Emotion regulation of self and others

Participants completed the Emotion Regulation of Others and Self scale (Niven, Totterdell, Stride, & Holman, 2011) to assess individual differences in the use of emotion regulation behaviours. Each of 19 items asks the extent to which people usually use strategies to regulate both their own and other people's affect on a scale ranging from 1 (*Not at all*) to 5 (*A great deal*). The four factors assessed: Extrinsic Affect-improving (e.g. ‘*I gave someone helpful advice to try to improve how they felt*’; *M* = 3.70, *SD* = 0.70, *α* = 0.84), Extrinsic Affect-worsening (e.g. ‘*I acted annoyed towards someone to try to make them feel worse*’; *M* = 1.43, *SD* = 0.44, *α* = 0.57), Intrinsic Affect-improving (e.g. ‘*I laughed to try to improve how I felt*’; *M* = 3.37, *SD* = 0.65, *α* = 0.72) and Intrinsic Affect-worsening (e.g. ‘*I expressed cynicism to try to make myself worse*’; *M* =1.79, *SD* = 0.90, *α* = 0.91). This scale has demonstrated appropriate psychometric properties, including yielding to the four-factor structure proposed, good correlations with existing measures of emotion-regulation strategies and convergence between self and peer reports (Niven et al., 2011).

##### Emotional intelligence

Participants completed an Emotional Intelligence scale that measures four facets of emotional intelligence (Law, Wong, & Song, 2004). The scale requires participants to rate their agreement to a list of 16 statements on a 6-point Likert format-scale from 1 (*Totally Disagree*) to 6 (*Totally Agree*). The measure is composed of the following four dimensions: Self-emotions Appraisal (e.g. ‘*I really understand what I feel*’; *M* = 4.49, *SD* = 0.67, *α* = 0.75), Others-emotions Appraisal (e.g. ‘*I am a good observer of others' emotions*’; *M* = 4.44, *SD* = 0.82, *α* = 0.86), Use of Emotions (e.g. ‘*I always tell myself I am a competent person*’; *M* = 4.33, *SD* = 0.89, *α* = 0.80) and Regulation of Emotion (e.g. ‘*I have good control of my own emotions*’; *M* = 4.20, *SD* = 1.11, *α* = 0.89).

##### Emotional expressivity

Participants also completed an emotional expressivity scale to assess behavioural emotional impulses (Gross and John (1997). This 16 item questionnaire uses a scale ranging from 1 (*Strongly disagree*) to 7 (*Strongly Agree*) to measure three facets of expressivity: The degree to which emotional tendencies are expressed behaviourally as Positive Expressivity (e.g. ‘*When I'm happy my feelings show*’; *M* = 5.33, *SD* = 1.32, *α* = 0.89), Negative Expressivity (e.g. ‘*It is difficult for me to hide my fear*’; *M* = 4.13, *SD* = 0.73, *α* = 0.78) and the strength of emotional response tendencies or Impulse Strength (e.g. ‘*I have strong emotions*’; *M* = 4.22, *SD* = 1.47, *α* = 0.89). These three facets have been shown to have associations with pertinent emotion scales and a robust relationship between self-reports and peer-rated reports.

##### Trait affect

Participants completed a short measure of trait affect (Thompson, 2007), which measures people's broad tendency to experience positive and negative mood. Participants are required to rate the extent to which they generally feel five Positive Trait Affect adjectives (e.g. *Inspired*; *M* = 3.44, *SD* = 0.58, *α* = 0.78) and five Negative Trait Affect adjectives (e.g. *Upset*; *M* = 2.00, *SD* = 0.49, *α* = 0.66) on a 5-point Likert format-scale from 1 (*Never*) to 5 (*Always*). This scale has previously demonstrated excellent test-retest reliability, satisfactory correlation with the positive and negative affect schedule (Watson, Clark, & Tellegen, 1988) and stability over time (Thompson, 2007).

##### Adult attachment style

Finally, chronic patterns of interaction were measured by including the adult attachment scale (Collins, 1996). Participants were instructed to think about current or previous relationships and respond in terms of how they generally feel in these relationships on a scale ranging from 1 (*Not at all characteristic of me*) to 5 (*Very characteristic of me*). This 18 item measure contains three subscales (each with six items) that measure: (i) the extent to which a person is uncomfortable with closeness and intimacy, that is, Discomfort with Closeness (e.g. ‘*I am somewhat uncomfortable being close to others*’; *M* = 2.59, *SD* = 0.37, *α* = 0.75); (ii) the extent to which individuals are uncomfortable depending on others, that is, Discomfort with Dependency (e.g. ‘*I find it difficult to allow myself to depend on others*’; *M* = 2.71, *SD* = 0.38, *α* = 0.72) and (iii) the extent to which someone is afraid about being rejected or abandoned by others, that is, Anxiety (e.g. ‘*I often worry that romantic partners don't really love me*’; *M* = 2.65, *SD* = 0.93, *α* = 0.86). Previous studies have shown that the three subscales consistently relate to their expected emotional and behavioural response patterns (Collins, 1996).

#### Stage 2: In situ speed-date measures

Participants completed a number of measures immediately after each date during the speed-dating event.

##### Affective presence

Participants reported the emotional experience that they felt with their recent partner for eight different emotions (*enthusiastic*, *happy*, *angry*, *bored*, *stressed*, *relaxed*, *calm* and *sad*) on a scale ranging from 1 (*Not at all*) to 5 (*A great deal*). These emotions were the same as those used by (Eisenkraft & Elfenbein, 2010) to measure affective presence. Following Eisenkraft and Elfenbein, we created two mean scale scores by reverse scoring *bored* to compute a dimension of positive affect that also included *enthusiastic* and *happy* (*α* = 0.81), and by reverse scoring *calm* and *relaxed* to compute a dimension of negative affect that also included *stressed* (*α* = 0.60). *Angry* and *sad* were excluded from the model because the response patterns for these emotions were invariant due to low endorsement of the items. Previous structural equation modelling analysis using this measure confirmed that the two factors defined by positive affect and negative affect provided a better fit to the data than a single global factor of positivity–negativity (Eisenkraft & Elfenbein, 2010). The presentation order of the emotion adjectives was randomized.

##### Perceived responsiveness

Participants also completed a short version of the perceived responsiveness scale (Reis et al., 2011) after each date, which evaluated the extent to which they perceived responsiveness from their partners. They answered four items concerning the extent to which their last date was attentive to them and reacted supportively to them (e.g. ‘…*listened to me*’; *M* = 4.42, *SD* = 1.42, *α* = 0.94), on a response scale ranging from 1 (*Not at all true*) to 7 (*Completely true*). The order of presentation of these items was randomized, as was the order of presentation of the affective presence and perceived responsiveness scales.

##### Romantic interest

Finally, a unique question asking whether participants would like to see each of their partners again was employed as a measure of romantic interest, in a dichotomous response scale (*i.e. yes*/*no*). Participants were told that a positive answer to this question would mean that their contact details would be passed on to dates who indicated a mutual liking.

#### Stage 3: End-of-event questionnaire

At the end of the event, participants completed a Big-5 personality measure using the 10-item Personality Inventory (Gosling, Rentfrow, & Swann, 2003), which has shown high reliability and convergent validity with other Big-5 measures (Gosling et al., 2003). Items assessing the dimensions of Agreeableness (*M* = 4.25, *SD* = 1.09), Conscientiousness (*M* = 4.45, *SD* = 1.96), Extraversion (*M* = 4.66, *SD* = 1.15), Emotional Stability (*M* = 4.41, *SD* = 1.28) and Openness to Experience (*M* = 4.77, *SD* = 1.01) were responded by using a scale from 1 (‘*Disagree strongly*’) to 7 (‘*Agree strongly*’).

### Data analysis strategy

Analysis of affective presence was conducted using the social relations model (SRM; Kenny, 1994; Kenny & La Voie, 1984). According to the SRM, emotions in dyadic interactions can be explained by three different sources of variability (plus error): the *actor effect* (the individual tendency to experience particular emotions, akin to trait affect), the *partner effect* (the individual tendency to be seen by others systematically in a certain way) and the *relationship effect* (the unique effect between two individuals). Affective presence is therefore a *partner effect* because it is the emotional experience elicited in others (*i.e*. how others tend to feel with an individual).

Multilevel modelling was employed to analyze the data because the speed-dating design meant that observations were non-independent due to the dyadic nature of the design. Thus, it is possible to distinguish three units of analysis: individuals (both as someone who rates or is rated by others; *i.e*. actor and partner), dyads and groups (in this case, the groups that we divided participants into), which give rise to a cross-classified data structure. The data analysis strategy included three stages. In the first stage, we estimated the SRM actor, partner and relationship variance without predictors using a random intercepts for actor, partner, dyad and group (Kenny & Livi, 2009). This procedure allowed us to determine the extent to which individuals differed in affective presence, and to establish whether these variations were significant across the whole sample (Kenny, Kashy, & Cook, 2006). For these analyses, we examined variance in each of the distinct emotion states we studied, as well as in mean positive affect and negative affect.

In the second stage, predictors of affective presence were estimated, this time focusing on mean positive and negative affective presence. To do so, it is important to consider that the data resulting from the speed-dating event were cross-classified because across dyads individuals were nested in multiple instances of interaction (Snijders & Kenny, 1999). Therefore, values of affective presence were firstly centred to group members' scores to control for differences between groups. This operation was repeated for perceived responsiveness. Individual differences variables were then introduced to the main dataset. Each case included the values of these variables for the partner, as well as for the actor, so that actor and partner effects could be addressed. For subsequent analysis, we kept the associations with the partner effects of each one. The individual difference variables were used one at a time to predict the affective presence scores, using random intercepts for actor, partner and dyad to address the cross-classified multilevel structure of the data. Disregard for this structure can lead to a greater probability of Type I errors and underestimate the standard error of fixed effects (Meyers & Beretvas, 2006). Hence, all fixed effects for the predictors of affective presence corresponded to estimations at the individual-level of analysis.

Finally, in the third stage, we performed a mediation analysis to establish whether the effect of perceived responsiveness on romantic interest was mediated by affective presence. Because the cross-classified structure of the dyadic data imposes some constraints on using contemporary meditational analysis techniques, we followed Baron and Kenny's steps for mediation analysis (Baron & Kenny, 1986; Judd & Kenny, 1981). We used generalized linear mixed models (GLMM) because romantic interest is a dichotomous variable, but using random intercepts for actor, partner, dyad and group to address the cross-classified multilevel structure of the data.

## Results

### Affective presence

To investigate affective presence, we first explored the consistency in partners' reports about how they felt after their dates. We found that partners' responses were correlated for positive affect, *ICC* (265) = 0.58, *p* < 0.01 and for negative affect, *ICC* (265) = 0.33, *p* < 0.01. This means that the affect scores within each dyad were related, for both positive affect and negative affect, which justifies the use of the SRM. The speed-dating event used only opposite gender dates so the dyad members could be distinguished from one another by gender, which has implications for the SRM model used (Kenny et al., 2006). Analyses demonstrated that a model based on undistinguishable dyads provided a better model fit compared with a distinguishable model, both for positive affect, *ΔAIC* = 7.0 and negative affect, *ΔAIC* = 7.4.^1^ This indicated that it was legitimate to pool results from men and women, which means that the asymmetric design can be considered for data analysis purposes as a symmetric block design (Lashley & Kenny, 1998). This gives approximately 80% statistical power for a partner effect estimate of 0.3 with three groups when *N* = 12 (Lashley & Kenny, 1998).

Table 1 shows the SRM variance partitioning both for positive affect and negative affect and for the discrete emotions that comprise these dimensions. The percentages in this table represent the intraclass correlation coefficient for actor effects, partner effects, relationship effects and error variance. Actor effects explained 30% of the variance in participants' positive affect, indicating that a significant amount of variance in people's positive affect after each date was explained by their own affective disposition, *ICC* = 30%, *Wald Z* = 3.38, *p* < 0.01. For negative affect, 37% of variance was explained by actor effects, *ICC* = 37%, *Wald Z* = 3.53, *p* <. 01.

**Table 1:** Social relations model of affective experience with speed dates

Emotion	*M*	*SD*	Actor variance (Trait affect)	Partner variance (Affective presence)	Relationship variance	Error
Positive affective presence						
Overall positive (*α* = 0.81)	3.73	0.84	30%**	15%**	5%	50%
Enthusiastic	3.21	1.13	32%**	10%*	4%	54%
Happy	3.38	1.02	37%**	8%*	2%	53%
Bored (reverse coded)	4.59	0.75	9%*	18%**	0%	73%
Negative affective presence						
Overall negative (*α* = 0.60)	2.11	0.66	37%**	0%	1%	62%
Stressed	1.30	0.69	29%**	0%	2%*	69%
Relaxed (reverse coded)	2.43	0.91	31%**	1%	0%	68%
Calm (reverse coded)	2.60	1.02	40%**	1%	0%	59%

*Note: N* = 268 observations. Affective experience ratings were made on a scale ranging from 1, *not at all*, to 5, *a great deal*.

* *p* < 0.05.

** *p* < 0.01.

The partner effects shown in the affective presence column in Table 1 describe the degree of consensus in the emotions that individuals elicited in others. A high percentage suggests that, across the sample, individuals elicited the same emotional experience in their partners. The results show that affective presence explained 15% of the variance in other people's positive affect, *Wald Z* = 2.67, *p* < 0.01. Moreover, affective presence variance for all discrete positive emotions exhibited significant results, explaining on average 13% of the variation in individuals' positive emotions. Variance in participants' boredom, in particular, was well-explained by affective presence, *ICC* = 18%, *Wald Z* = 2.64, *p* < 0.01. However, affective presence was not significant for negative affect, explaining less than 1% of the variance. Similarly, none of the discrete negative emotion variables showed significant partner effects. Negative affective presence was therefore excluded from subsequent analyses because it did not show significant results for partner variance.

### Interpersonal consequences of affective presence

Does affective presence predict romantic interest? A GLMM analysis—using a logit link function—for the effect of positive affective presence upon romantic interest (as indicated by the partner's desire to see the person again) demonstrated that positive affective presence was significantly associated with the partner's romantic interest, *b* = 1.15, *p* < 0.01, with an effect estimated to lie between 0.73 and 2.30. In other words, one unit increase in positive affective presence results in about a 29% increase in the probability to receive a ‘yes’ after each date. The variance explained by fixed factor was equal to *R^2^_GLMM(m)_* = 0.22, and the variance explained by the entire model (fixed and random effect) was equal to *R^2^_GLMM(c)_* = 0.65, reflecting that much of the data variability exists in random effects (Nakagawa & Schielzeth, 2013), which means that other variables, beyond positive affective presence, may also exert significant influence over romantic interest during dyadic interactions.

There was also evidence that positive affective presence mediated the relationship between perceived responsiveness and romantic interest. Linear mixed model analysis revealed that perceived responsiveness predicted positive affective presence, *b* = 0.47, *t*(224) = 16.70, *p* < 0.01. Moreover, GLMM showed that perceived responsiveness was significantly associated to romantic interest, *b* = 0.90, *p* < 0.01, with a direct effect estimated to lie between 0.56 and 1.24; *R^2^_GLMM(m)_* = 0.09; *R^2^_GLMM(c)_* = 0.59. When positive affective presence was included in the later model, the effect of perceived responsiveness was no longer significant, *b* = 0.33 (*SE* = 0.23), *p* = 0.16, while positive affective presence predicted romantic interest, *b* = 1.52 (*SE* = 0.40), *p* < 0.01; *R^2^_GLMM(m)_* = 0.10; *R^2^_GLMM(c)_* = 0.61 (see Figure 2). A Sobel test confirmed that positive affective presence significantly mediated the effect of perceived responsiveness on romantic interest, *z^´^* = 3.69 (0.19), *p* < 0.01. Together these results support hypotheses 1 and 2. However, it is important to mention that because the partner effect was found significant only for positive affective presence, these results were only supported with respect to positive affective presence and not for negative affective presence.

### Correlates of affective presence

An exploratory analysis investigated the correlates of positive affective presence.^2^ The results are summarized in Table 2. No significant differences were found for positive affective presence by study level (*i.e*. postgraduate vs undergraduate students), language (*i.e*. native English speakers vs non-native English speakers) or gender. However, some predictors (e.g. emotional expressivity) showed significant differences for gender, so analysis controlled for gender when this applied.

**Table 2:** Associations between affective presence and individual difference variables

	Positive Affective Presence		
	*β*	*Std. Error*	*Χ^2^_Change_*
Emotion Regulation of Others and Self			42.25***
Extrinsic improving	0.05	0.11	
Extrinsic worsening	−0.01	0.16	
Intrinsic improving	0.22*	0.12	
Intrinsic worsening	−0.11	0.08	
Emotional intelligence			61.48***
Self-Emotions appraisal	−0.18†	0.12	
Others-Emotions appraisal	0.23*	0.11	
Use of Emotion	0.17†	0.09	
Regulation of emotion	0.12	0.07	
Emotional Expressivity			58.70***
Positive expressivity	0.07	0.07	
Negative expressivity	0.27*	0.08	
Impulse strength	0.05	0.06	
Trait affect			36.47***
Positive trait affect	0.06	0.15	
Negative trait affect	−0.12	0.18	
Adult Attachment			64.05***
Discomfort with closeness	−0.02	0.15	
Discomfort with dependency	−0.32*	0.14	
Anxiety	0.24**	0.07	
Big Five factors			15.81*
Extraversion	0.27**	0.06	
Agreeableness	0.19*	0.06	
Conscientiousness	0.12	0.07	
Openness to experience	0.03	0.07	
Emotional Stability	0.02	0.06	
Perceived responsiveness	0.77***	0.03	178.18***

*Note: N* = 40 participants. Standardised estimators reported. *χ^2^_Change_* based on the difference between the intercept only model (*df*. = 6) and the model with predictors.

† *p* < 0.10.

* *p* < 0.05.

** *p* < 0.01.

*** *p* < 0.001.

#### Emotion skills and dispositions

The first group of predictor variables corresponds to individual differences in emotion skills and emotion dispositions. Of the variables relating to use of emotion regulation strategies, only intrinsic affect-improving strategy use was significantly associated with positive affective presence, *b* = 0.27, *t*(38) = 2.24, *p* < 0.05, suggesting that individuals who deliberately improve their own feelings were more likely to elicit positive emotions in their dates. Of the emotional intelligence factors, only the ability to perceive and understand others' emotions (others' emotion appraisal) was positively associated with positive affective presence, *b* = 0.24, *t*(38) = 2.20, *p* < 0.05, such that individuals who were more skilled in this respect were more likely to elicit positive emotional reactions in their dates. Regarding emotional expressivity, only negative emotional expressivity was associated with positive affective presence, *b* = 0.18, *t*(38) = 2.22, *p* < 0.05; individuals who easily disclose their negative emotions were more likely to elicit positive emotional reactions in their dates. Finally, neither positive nor negative trait affects were significantly associated with positive affective presence.

#### Attachment style and personality traits

The second group of variables tested as predictors of affective presence were individual differences in attachment style and personality traits. Chronic activation patterns measured by adult attachment dimensions showed that individuals high on discomfort with dependency exhibited less positive affective presence, *b* = −0.35, *t*(36) = −2.43, *p* < 0.05. Conversely, individuals high in anxious attachment had higher positive affective presence, *b* = 0.21, *t*(37) = 2.80, *p* < 0.01. Of the Big-5 personality factors, extraversion, *b* = 0.19, *t*(39) = 3.06, *p* < 0.01 and agreeableness, *b* = 0.14, *t*(38) = 2.30, *p* < 0.05, were associated with positive affective presence. Thus, individuals who self-reported as being more talkative or energetic, as well as those self-described as friendlier or socially-driven, tended to elicit more positive affect in others. The other measures of attachment and personality did not show significant associations with positive affective presence.

## Discussion

Personality research has traditionally used differences between people's personal emotional experiences as a means of identifying individual differences in emotional temperament. In contrast, the present study has examined whether differences in the emotions that people consistently elicit in others—known as affective presence—is an alternative and influential emotion-related individual difference. This approach is consistent with recent calls to integrate personality and interpersonal relationships within a unified framework (Back *et al*., 2011; Graber et al., 2011) and with contemporary accounts about the informational properties of emotions (Forgas, 1994; Schwarz & Clore, 1983; Van Kleef, 2009). In the present research, we pursued this approach by studying affective presence in the context of romantic interactions during a speed-dating event.

The research makes three main contributions to the understanding of affective presence. Firstly, building on prior work on emotion and attraction (e.g. Kashdan et al., 2007; Mehrabian & Blum, 1997; Sunnafrank, 1986; Tracy & Beall, 2011), our findings suggest that affective presence is an important predictor of romantic interest. In our study, the dates of people possessing greater positive affective presence were more likely to want to see them again. Thus, eliciting positive emotions in one's interaction partner may serve an important social function of promoting romantic attraction. The behavioral nature of our measure of romantic interest, in which people stated an interest in meeting another person again and were prepared to reveal their contact details to that person, adds weight to this claim. Participants in this study did not simply rate the likeability of each partner; instead, they expressed their intention to initiate a real relationship by acceding to reveal their personal details. It would be interesting to investigate whether affective presence influences the development as well as initiation of interpersonal relationships.

Affective presence was also shown to be a mediator in the relationship between the perceived responsiveness of a dating partner and romantic interest in that person. It seems, therefore, that being responsive instigates the experience of positive affect in others, and this experience is a proximal predictor of romantic interest under the condition that the emotional impact is generalized across multiple partners. It is interesting to speculate that this pathway could occur either because responsiveness influences the deployment of the personal repertoire necessary to elicit positive affect in others (*i.e*. by engaging in responsive interactions, people high in positive affective presence can deploy their personal resources to elicit positive affect in others) or because responsiveness heightens the perception of relevant affective characteristics in some partners, including the tendency to elicit consistently positive affect in others which assists in determining romantic interest.

Secondly, our findings provide insight into some of the personal characteristics that relate to having a positive affective presence. With respect to emotion skills and dispositions, we found that individuals who typically try to improve their own emotions and those who understand the emotional experience of others were more likely to consistently make others feel more positive emotions. Our findings indicate that people who consistently elicit positive emotions in others are not necessarily the same as those who experience more pleasant affect themselves. Interestingly, with respect to individuals’ tendencies for emotional expression, only negative expressivity predicted positive affective presence. Though this result was surprising, previous research has shown that the expression of negative emotions during short interactions does not differ significantly from the expression of positive emotions in predicting perceived partner rapport, and in general, the expression of negative emotions lead to better perceived partner rapport and liking than suppressing such emotions (Butler et al., 2003).

The utility of unpleasant emotions in developing interpersonal rapport also helps explain the positive relationship that was found between anxious attachment and positive affective presence. Anxiety has been associated with easier self-disclosure (Keelan, Dion, & Dion, 1998; Mikulincer & Nachshon, 1991), which in turn, can lead to more positive evaluations from others (Collins & Miller, 1994), so individuals high in anxious attachment may have been more self-disclosing to elicit positive evaluations from partners and thereby avoid the threat of rejection.

Regarding personality traits, agreeableness and extraversion were positively associated with positive affective presence. Presumably, agreeable people engage easily in using considerate behaviours, and as a consequence, produce pleasant affective responses in others (Moskowitz & Côté, 1995). However, a positive relationship between extraversion and *positive* affective presence was unexpected, because Eisenkraft and Elfenbein (2010) found that extraversion was positively related with *negative* affective presence. The explanation may lie in the different nature of the interpersonal interactions in the two studies. In the present study, the participants chose to engage in a social event that was intended to be pleasurable; whereas in the earlier study, the participants were interacting in task-focused groups. Evidence from the behavioural concordance model (Côté & Moskowitz, 1998) suggests that extraverts experience positive affect when they are involved in pleasant interactions. Accordingly, it is plausible that extraverts felt positive emotions because they were engaged in pleasant interactions during the speed-dating event and therefore elicited congruent pleasant emotions in others.

Overall, the evidence concerning the correlates of affective presence indicates that positive affective presence arises from other-oriented features of the self, emphasizing the interpersonal nature of this personality characteristic. The evidence presented here indicates that individual variables that enable people to understand others' emotions and express their own emotions relate with affective presence. However, a certain degree of personal emotional management—as demonstrated by the association between positive affective presence and the use of intrinsic emotion regulation strategies—also seems to be necessary in order to consistently elicit positive affect in others.

Thirdly, our study provided a constructive replication of affective presence. Affective presence has thus far only been demonstrated in a single study, conducted in a work context (Eisenkraft & Elfenbein, 2010). In the present study, we found evidence that people also consistently elicit positive emotion in others in a romantic context (speed-dating). Furthermore, we have addressed some limitations of the previous study. In their original study, Eisenkraft and Elfenbein studied affective presence by taking a single measure of affect to cover a month's worth of interactions. A limitation of this approach is that a range of other interpersonal or group processes not controlled for during this month could also have explained differences in the emotions reported by participants. In the present study, to address this issue, we studied a series of one-off dyadic interactions of a short duration (4 minutes), collecting measures of affect immediately after each interaction.

It is noteworthy that only positive affective presence was observed in the present study, whereas in Eisenkraft and Elfenbein's (2010) original study negative affective presence was actually found to explain more variance in other people's feelings. Several theoretical accounts support the idea that interpersonal situations are organized around two basic dimensions: affiliation (e.g. close and warm interactions) and social dominance (e.g. competence and commanding behaviours) (Benjamin, 1974; Cuddy, Fiske, & Glick, 2008; Wiggins, 1979). Previous research on social perception has demonstrated that warmth and competence are in general negatively related (Judd, James-Hawkins, Yzerbyt, & Kashima, 2005) and can give rise to different emotional experiences (Fiske, Cuddy, Glick, & Xu, 2002). Therefore, it may be that interactions characterized by affiliation (e.g. romantic dating) may make indicators of positive affective presence more salient to individuals, whereas interactions characterized by task orientation (e.g. group projects) may make indicators of negative affective presence more salient. Individual differences in affective presence might therefore be best understood by using a person-situation personality profile involving coherent behavioural patterns in response to similar situations (Mischel, 2004; Mischel & Shoda, 1995).

In addition to contributions regarding affective presence, the study presented in this paper also contributes more broadly to understand the relationship between emotion and personality. Our results demonstrated that affective presence was not related to trait positive or negative affect. Thus, beyond individual tendencies to experience positive and negative affect in general, there also exists an individual difference that lies in the emotional experiences elicited in others. The phenomenon of affective presence emphasizes that individual dispositions that create invariances in others' behavior may be considered within the construct of emotion personality. Consistency of personal behaviours has been considered as a key criteria for identifying individual differences (McAdams, 1997). Individual differences in affective presence further this notion by indicating that consistency in others' affective reactions to a person may designate an individual difference. Moreover, affective presence can be distinguished from previous attempts to identify intraindividual patterns using personality judgments (e.g. Funder, 1995). In the judgment approach, the extent to which informants agree about someone's personality traits is usually seen as evidence of consistency of personality characteristics (McCrae & Costa, 1987). However, affective presence refers to the effects of an individual upon others' emotional *experience*, which does not have to correspond to the individual's own characteristic emotional experience or to other people's judgment of that characteristic.

### Limitations and future research

The design of the present study had a number of strengths, notably the speed-dating design in which we tested consistency in affective presence during a large number of interactions and the use of other-reported data (regarding participants' affective presence), which addresses concerns of common-method variance often associated with the use of self-reported data. Nevertheless, some limitations remain. Firstly, we focused on the distinction between positive and negative affective presence. Like most emotion-related concepts, the expression of affective presence probably has features beyond the simple positive/negative distinction proposed here. The fact that some particular affective presence reactions showed stronger effects than the corresponding actor effects (e.g. boredom) suggests that a closer look is necessary to understand the *presence* of particular emotions under different circumstances. For example, boredom could be a very salient affective presence reaction in romantic interactions, but might not have the same consequences in other domains.

A second limitation is the cross-sectional nature of the data collection after each date. Because each date was so short, it was only possible to collect data about affective presence, perceived responsiveness and romantic interest in the same questionnaire. This means that it is not possible to determine causality (e.g. does affective presence cause romantic interest or does romantic interest influence partners' affect during the date?), which is particularly salient for the mediation analysis, in which a causal chain is inferred. Future studies could therefore consider the potential for manipulating affective presence to enhance causal inference and explore the potential malleability of affective presence for intervention purposes.

Another limitation of the present study is its power. Although SRM analysis benefits from more observations per person, meaning that increasing the number of observations for the present study to 6–7 compared with the 3–4 observations per person observed in Eisenkraft and Elfenbein's study represents an improvement, our analysis is limited to just three groups of participants. Furthermore, our reported correlates of affective presence are associations at the individual-level of analysis, and our sample of 40 participants represents a modest number of subjects to support conclusive evidence. Hence, these results need to be interpreted with caution.

A final limitation regards our lack of a measure of physical attractiveness. In our study, affective presence was shown to have an important role in the initiation of romantic relationships. However, physical attractiveness has pervasive effects on interpersonal attraction (Back *et al*., 2011; Hatfield & Sprecher, 1986) and might have influenced romantic interest and potentially also the emotions elicited in others. Future studies should investigate how affective presence and physical attractiveness relate.

### Conclusion

The evidence for affective presence as a phenomenon demonstrates that some individual differences can be found outside the person. Notably, positive affective presence predicted whether a person would be sufficiently liked by others to obtain second dates, and mediated the relationships between a documented source of likeability (*i.e*. perceived responsiveness) and romantic interest. These results emphasize the importance of taking into account the role of affect when conducting research on interpersonal relationships. This role is likely to amount to more than just provision of an undifferentiated arousal pattern because affective presence appears to vary across emotions and setting. Aside from producing a replication of a new phenomenon, this study has also provided further evidence concerning the personality correlates of affective presence, which include aspects of trait emotion-regulation, emotion-understanding, emotional-expressiveness and attachment style, but may exclude trait affect. Ultimately, the study of affective presence raises the idea that what emotionally distinguishes one individual from another lies in part in the emotional consequences of their behaviours on others.

## Footnotes

1 Model estimation was repeated for each of the individual emotion adjectives yielding similar model fits.

2 For the sake of simplicity, only the associations between positive affective presence overall and the predictor variables are reported; nonetheless, the analysis of the individual emotion adjectives led to similar result patterns.
